# Preparation of Luminescent Thermotropic Liquid Crystal from Benzodiathiazole Derivatives

**DOI:** 10.3390/ma12121919

**Published:** 2019-06-14

**Authors:** Yuchen Feng, Huijuan Yu, Dexun Xie, Yi Zhu, Xinhao Zhong, Chengjun Pan, Guang Shao

**Affiliations:** 1 School of Chemistry, Sun Yat-sen University, Guangzhou 510275, China; Fyuchen96@163.com (Y.F.); yhjuan@mail.sysu.edu.cn (H.Y.); 2 Shenzhen Research Institute, Sun Yat-sen University, Shenzhen 518057, China; 3 Shenzhen Key Laboratory of Polymer Science and Technology, College of Materials Science and Engineering, Shenzhen University, Shenzhen 518060, China; zhuyizhuyi913913@163.com (Y.Z.); s1920294@s.tsukuba.ac.jp (X.Z.); pancj@szu.edu.cn (C.P.)

**Keywords:** solid-state emission, luminescent liquid crystal materials, Benzo[c][1,2,5]thiadiazole

## Abstract

Luminescent liquid crystal materials (LLCMs) have been a hot research topic in the field of fluorescent materials. In this study, we successfully designed and synthesized an intense fluorescence thermotropic liquid crystal material with a fluorescence quantum yield (*Φ*) of 0.26 in the solid state. Moreover, the alkyl chain attached to the terminus of the chromophore was able to promote the stability of electrochemical and thermal properties, which was beneficial to the device fabrication reproducibility and stability of the device performance.

## 1. Introduction

Liquid crystal materials (LCMs) have drawn significant attention due to their wide applications in organic optoelectronic devices, such as optical information displays and storage devices [1,2,3]. However, most of the liquid crystal materials investigated are not fluorescent materials and thus limit the application of liquid crystal displays. Therefore, luminescent liquid crystal materials (LLCMs) with intrinsic fluorescence properties and an organized structure are highly desired, and have been rapidly developed and used in anisotropic light-emitting diodes, polarized organic lasers, information storage, and sensors [1,2,3,4,5,6,7,8,9,10]. 

Despite the promising features of fluorescent LCMs, the incorporation of luminescent functional groups into LCMs while retaining the mesomorphic properties is still challenging, because most of the fluorescent molecules are only highly emissive in a solution, and their fluorescence quantum yields are greatly decreased from the solution to the condensed state [11,12,13,14]. Therefore, a new approach is necessary to synergistically generate efficient luminescence in the mesophase. Thus, much research has been devoted to the design and synthesis of new florescent materials that can emit intense fluorescence in the solid state, such as a series of solid-state emissive organic molecules developed by Tang et al., which exhibit so-called aggregation-induced emissions (AIE) [15]. By taking the benefit of the aggregation-induced emissions and the organization of liquid crystal, a variety of LCMs with AIE properties have been developed and show promising applications in the field of optoelectronic materials [1,2,3].

Red-emitting materials, especially those where the emission wavelength includes the deep-red and near-infrared radiation (NIR) range, have attracted great attention in the fields of materials science, chemistry, and biology [16,17,18]; however, materials that can emit a red light generally require a narrow Highest Occupied Molecular Orbital (HOMO) and Lowest Unoccupied Molecular Orbital (LUMO) band gap, and thus, most of the reported strategies to generate red emission are directed to introducing electron-donating and electron-accepting groups to the termini of π-conjugated moieties [19]. However, molecules with such types of structure generally have a strong tendency to form π–π stacking in the solid state, resulting in luminescence quenching caused by the dipole-dipole interactions from the donor and acceptor segments; therefore, most of the red-emitting materials are susceptible to concentration quenching and become either weakly luminescent or even not emissive at all in the condensed state [20]. In this case, the development of red-emitting materials with high fluorescence efficiency (*Φ*) and liquid crystal properties is highly desired, although, to the best of our knowledge, only a few examples of LCMs that emit a red light have been reported [16,17,18]. Huang et al. synthesized a red-emitting spirobifluorene containing a diphenylamine group as donor and a dicyanoethenyl group as acceptor in the termini. This type of molecule shows an emission maximum at 644 nm with a quantum yield of 0.33 [19]. In this molecular design, the nonplanar structure of the diphenylamino moiety and the spiro framework contribute to the suppression of π–π stacking of the dye in the solid state, and the choice of a dicyanoethenyl moiety as an acceptor is the key to generating red emission. Osuka et al. introduced four hexyl groups to the 2, 5, 8, and 11 positions of perylene tetracarboxylic acid bisimide, resulting in a highly emissive dye with an emission maximum of 635 nm and fluorescence efficiency of 0.59. These results are remarkable compared to the perylene tetracarboxylic acid bisimide without the hexyl groups (*Φ*_powder_ = 0.07) [21]. Cocchi et al. reported a NIR excimer-based organic light-emitting diode with a high efficiency of 14.5% ± 0.2% photon/electron that exhibits exclusive NIR excimer emission peaking at a wavelength of 700 nm [22]. Vollbrecht et al. quantified the relationship between the red-shift and interplanar distance by investigating perylene-, phenanthroperylene- and dinaphthocoronene-esters. According to their research, the energy difference (ΔE_Shift_) will increase with a smaller value of interplanar distance [23,24]. Recently, Anthony et al. developed a triphenylamine and N-methyl barbituric acid/indane dione-based donor–acceptor derivative with red-emitting material and quantum yields of 0.14 to 0.41 [25]. Park et al. developed a distyrylbenzene-based D-A-D-A-D red/near-infrared-emitting material with a quantum efficiency of 0.36 [26]. Zhang et al. developed organoboron-based compounds with NIR emission and quantum efficiencies of 0.21–0.30 [27]. Tang et al. developed an N, N-dimethyl-attached fumaronitrile-based (D–A) molecule that had a quantum efficiency of 0.27 [28]. These examples indicate that intense red-emitting materials could be generated through a rational molecular design, but to the best of our knowledge, materials that exhibit deep-red emission with wavelengths close to 700 nm are still rare.

In this research, we synthesized 4,7-bis (thiophen-2-yl) benzo[c][1,2,5]thiadiazole derivative (BTC0) with a quantum efficiency of 0.1 in the solid state with a deep-red emission of 689 nm. Interestingly, a simple introduction of hexyl groups to the terminus of 4,7-bis(thiophen-2-yl)benzo[c][1,2,5]thiadiazole derivative (BTC6) using a palladium-catalyzed Stille coupling reaction was able to generate an intense deep-red emission at approximately 689 nm in the solid state with a rather high fluorescence quantum yield of 0.26. The attached flexible aliphatic hexyl chains provided the molecules with smectic liquid crystal features upon heating.

## 2. Results and Discussion

### 2.1. Molecular Design and Synthesis

**Figure 1:**
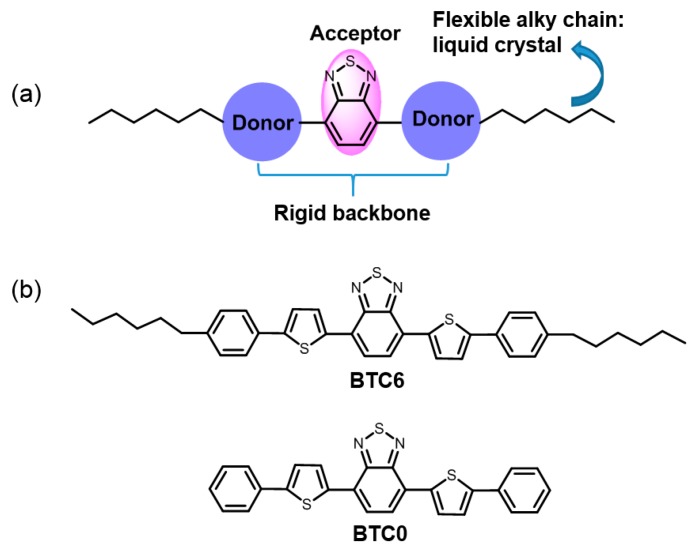
Molecular design of D-π-A molecules based on benzodiathiazole moiety (**a**) and chemical structures of the model compounds 4,7-bis (thiophen-2-yl) benzo[c][1,2,5]thiadiazole derivative (BTC0) and 4,7-bis(thiophen-2-yl)benzo[c][1,2,5]thiadiazole derivative with a simple introduction of hexyl groups (BTC6) (**b**).

**Scheme 1:**
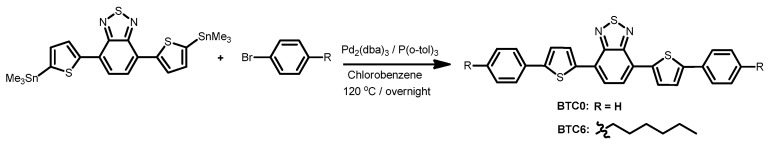
Synthetic scheme of BTC0 and BTC6 chromophores.

Due to the electron-deficient feature of Benzo[c][1,2,5]thiadiazole (BT), it has been widely used to construct a variety of donor-acceptor (D-A) systems with appropriate donor segments in π-conjugated small molecules or polymers. In this research, as shown in Figure 1, thiophene and benzene segments were chosen as donor, and a Benzo[c][1,2,5]thiadiazole (BT) part was chosen as acceptor to construct a simple D-A system. Meanwhile, the flexible hexyl chain was attached to the terminus of the rigid backbone of the D-π-A core (BTC6) to generate a liquid crystal feature. A naked D-π-A core (BTC0) without a flexible alkyl chain was also designed and synthesized for comparison. Both BTC6 and BTC0 were synthesized via Stille coupling with excellent yields (Scheme 1).

### 2.2. The Photophysical Properties of BTC0 and BTC6 in Solution

**Figure 2:**
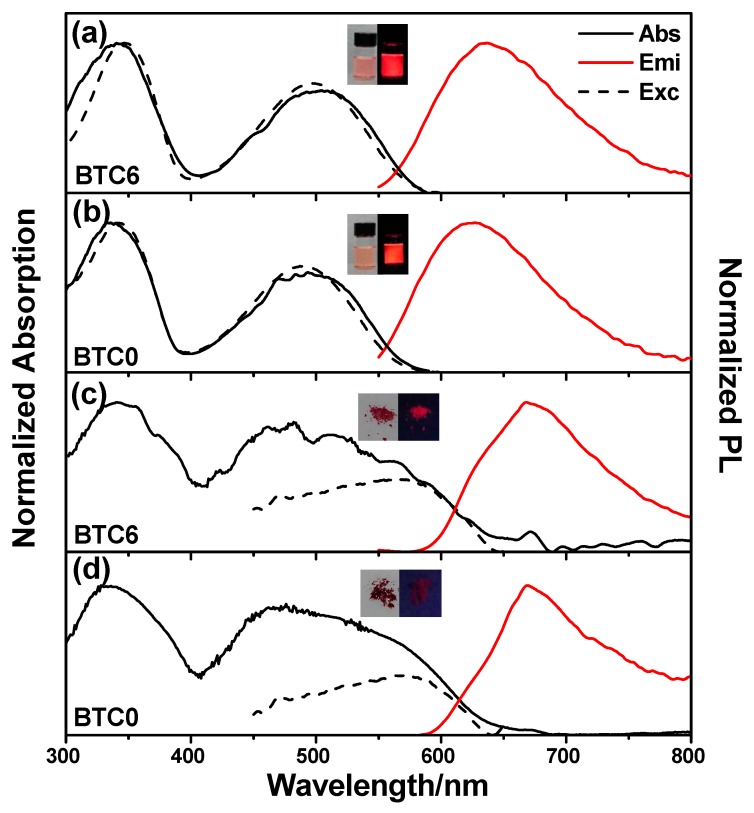
The absorption, emission, and excitation spectra for BTC6 in a chloroform solution (1.0 × 10^−5^ M) (**a**) and in the solid state (**c**). The absorption, emission, and excitation spectra for BTC0 in a chloroform solution (1.0 × 10^−5^ M) (**b**) and in the solid state (**d**).

The UV-vis, photoluminescence (PL) and excitation spectra are shown in Figure 2. In Table 1, the wavelength of absorption and emission maximum, extinction coefficient (ε), Stokes shift, quantum yield (*Φ*_F_), lifetime (*τ*_f_), radiative decay rate constant (*k*_r_), and nonradiative decay rate constant (*k*_nr_) are summarized. The results showed that BTC6 and BTC0 had multiple absorption bands in both the solution and the solid state, with one band approximately 500 nm assigned to the intramolecular charge transfer from the donor to the acceptor core and the other band at approximately 350 nm assigned to the π–π* transition. In addition, the solvatochromic effect of the PL spectra was observed (Figure S1c,d, and Table S1). When the solvent polarity was increased gradually from low-polarity hexane to high-polarity DMSO, both BTC6 and BTC0 exhibited a large redshift of approximately 70 nm, which indicated that the first singlet excited state (S_1_) of the dye possessed a strong intramolecular charge transfer characteristic.

Comparing the spectra of BTC6 and BTC0 in the solution (Figure 2a,b), they did not exhibit marked differences with respect to spectral shape and the wavelengths of absorption and emission maximum. Additionally, the values of the Stokes shift, quantum yield (*Φ*_F_), lifetime <*τ*_f_>, radiative decay rate constant (k_n_), and nonradiative decay rate constant (k_nr_) were very similar (Table 1, and Figure S2a), indicative of the similar optical properties of the compounds in the solution. We presumed that the molecules existed in a practically monodispersed isolated state in dilute solution and that the length of the alkyl chain did not affect the electronic transition; therefore, the compounds had similar optical properties in the dilute solution.

By contrast, the two compounds showed a different optical performance in the solid state. Obviously, BTC0 had a smaller quantum yield and lifetime (*Φ*_F_ = 0.10, <*τ*_f_> = 1.97 ns) compared to that of BTC6 (*Φ*_F_ = 0.26, <*τ*_f_> = 7.17 ns). Furthermore, the k_r_ value of BTC0 (5.07 × 10^7^ s^−1^) was 1.3 times that of BTC6 (3.63 × 10^7^ s^−1^), while the k_nr_ value of BTC0 (4.57 × 10^8^ s^−1^) was nearly 5 times that of BTC6 (1.03 × 10^8^ s^−1^). The significant enhancement of the k_nr_ in BTC0 was ascribed to the acceleration of the nonradiative deactivation channel arising from π−π interactions. 

### 2.3. PXRD Patterns of the Crystalline Powders of BTC0 and BTC6

**Figure 3:**
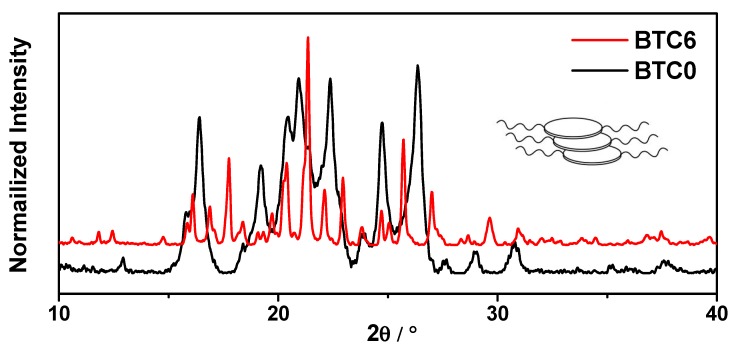
X-ray diffraction patterns of BTC0 and BTC6 and model of *J*-type aggregates.

According to the X-ray diffraction (XRD) patterns (Figure 3), the results showed that the distance between adjacent molecules of BTC6 was 4.16 Å, which is greater than the typical intermolecular π–π interactions of 3.80 Å. Thus, BTC6 avoided the quenching of fluorescence in the solid state. Moreover, BTC0 showed a closer distance (3.37 and 3.59 Å) within the range of intermolecular π–π stacking, thus making the intermolecular π–π interactions sufficiently strong to quench the fluorescence in the solid state. Apparently, the alkyl chains of the molecule trigger showed a different aggregation, as shown by the distinct differences in optical properties. BTC0 without alkyl chains had a strong interaction among the coupled cores to induce a short distance stacking of 3.37 and 3.59 Å. BTC6 with alkyl chains had van der Waals interactions among the alkyl side chains, which provided force to counterbalance against the interactions between the coupled cores, resulting in a longer distance of 4.16 Å. In summary, we can conclude that the existence of an alkyl chain induced the enhancement of optical properties in the solid state.

In addition, we determined the types of molecular aggregation. Two main species of dye aggregates are distinguished in the literature: For *H*-aggregates, the absorption maximum of the aggregate is blue-shifted with respect to the isolated chromophore and fluorescence of the isolated chromophore is lost in the aggregate; for *J*-aggregates, the absorption and emission maxima are red-shifted and no quenching occurs in the aggregate state [29,30,31]. The common structural model for such aggregates is based on the longitudinal offset of the dye molecules, which is sometimes described as the deck-of-cards model (Figure 3), in which the cards represent the molecules stacked on top of each other [32,33]. By comparing the absorption and excitation spectra, the excitation at approximately 570 nm was the cause of emission and was red-shifted with respect to the isolated chromophore, indicating the formation of *J*-type aggregates.

### 2.4. Electrochemical Properties of BTC0 and BTC6

Theoretical calculations demonstrated that the molecules had LUMO and HOMO energy levels of –2.6 and –5.0 eV, respectively (Figure S3). To evaluate the compound energy levels experimentally, we investigated their electrochemical properties using cyclic voltammetry (CV) and differential pulse voltammetry (DPV) with ferrocene used as an internal standard. The electrochemical HOMO-LUMO band gaps are presented in Table 2. These results clearly indicated that the alkyl chain slightly affected the HOMO-LUMO levels and the band gap of the dyes.

**Figure 4:**
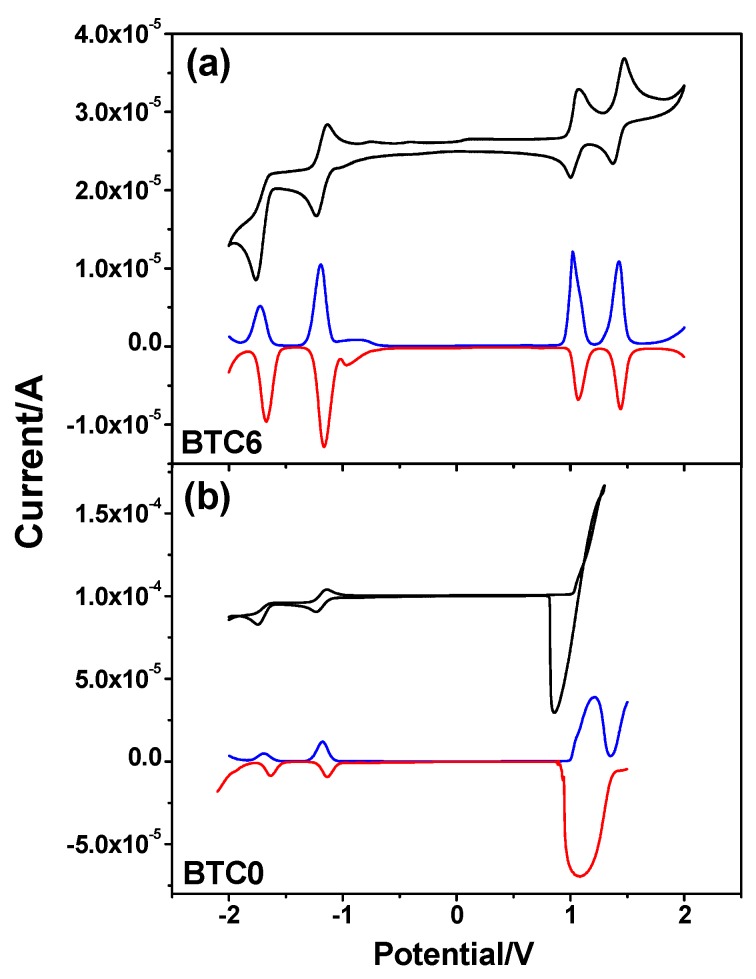
Cyclic voltammetry (CV) of BTC6 (**a**) and BTC0 (**b**) in a dichloromethane solution containing the electrolyte 0.1 M NBu_4_PF_6_. The compounds were measured at a concentration of 10^-3^ M in the electrolyte solution, with an Ag wire reference electrode and a Pt disk working electrode (scan rate = 100 mV s^−1^). Ferrocene was used as an internal standard. Differential pulse voltammetry (DPV) was measured using the same electrolyte with 50 ms pulse width, 50 mV amplitude, 200 ms pulse period, and 4 mV potential increment.

As shown in Figure 4, BTC0 and BTC6 exhibited a similar reversible reduction process. However, they showed a different oxidation process: BTC6 had two oxidation potentials, while BTC0 just had one oxidation potential. This characteristic can be explained by the molecular structure of BTC6. In BTC6, the active para-hydrogen in benzene was replaced by an inactive alkyl chain, which provided a protective effect and caused BTC6 to have a high resistance toward decomposition reactions, such as dimerization. Hence, BTC6 had a second oxidation potential, while BTC0 underwent decomposition reactions at that corresponding potential (such as electropolymerization, Figure S4). In summary, we can conclude that the alkyl chain can enhance the electrochemical stability of the molecule by replacing the active groups. Usually, the wider reversible redox potential range is favorable for the processes of device fabrication and other kinds of applications (organic light emitting diodes, sensors, etc.).

### 2.5. Thermal Properties and the Liquid Crystalline Phase

The thermal properties of the two molecules were investigated by thermogravimetric analysis (TGA) and differential scanning calorimetry (DSC). The TGA analysis revealed that the 5% weight loss temperatures (*T*_d_) of BTC6 and BTC0 were 366 and 314 °C, respectively (Figure S5). This result indicated that BTC6 had a better thermal stability, which can be attributed to the attached hexyl chain on BTC6, which can absorb more heat than BTC0.

**Figure 5:**
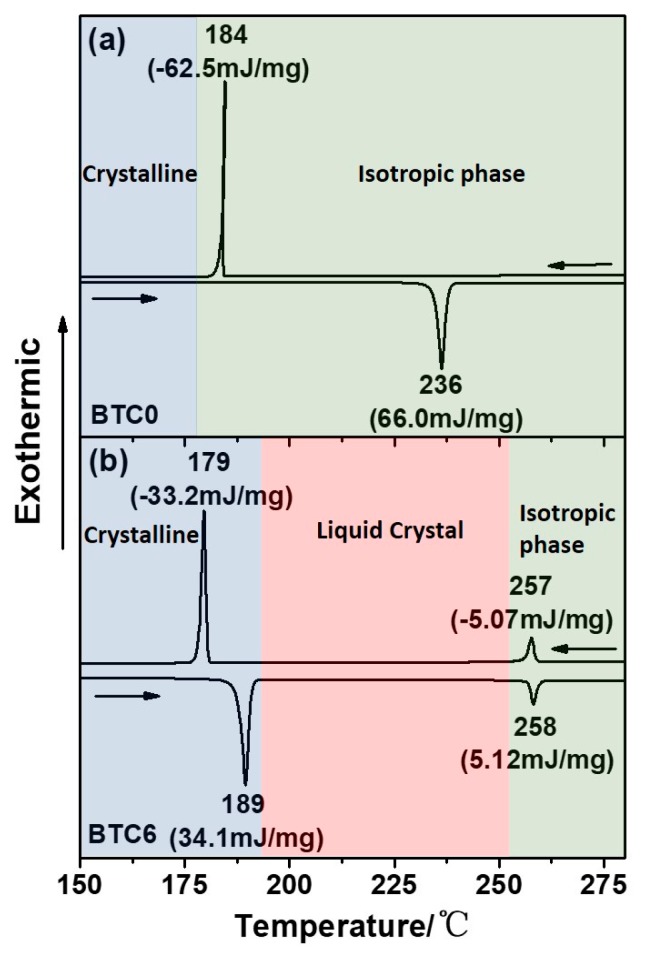
Differential scanning calorimetry (DSC) spectra of BTC0 (**a**) and BTC6 (**b**). The traces were measured at 20 °C/min and the second heat-cooling cycles were used.

**Figure 6:**
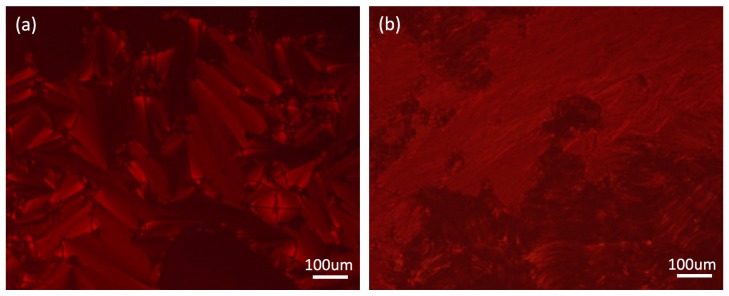
Fan-shaped texture (**a**) and crystalline phase (**b**) of BTC6 observed by a polarized microscope at 222.3 °C and 170.1 °C, respectively, during the cooling process.

In the heating process, BTC0 showed crystal phases below 236 °C and an isotropic liquid phase above that temperature (Figure 5a). In the cooling process, reverse phase transitions were observed. Interestingly, BTC6 showed a mesophase between the crystal phase and the isotropic liquid phase (Figure 5b). In Figure 6a, the texture of the mesophase observed with a polarized microscope (POM) is shown, namely, a fan-shaped texture, which is characteristic of the smectic phase [35,36,37]. The smectic phase was reasonable because the enthalpy changes in the phase transition from the crystal phase to the mesophase (34.1 J/g) were larger than those from the mesophase to the isotropic liquid phase (5.12 J/g). This result means that in the mesophase, the crystal structure was considerably disrupted, but the ordered structure remained. This characteristic can be explained by the molecular structure of BTC6 with a rigid central core and flexible side chains.

## 3. Experimental Section

### 3.1. Measurements

Air and water sensitive synthetic manipulations were performed under a nitrogen atmosphere using standard Schlenk techniques. All chemicals were purchased from Annaiji (Shanghai, China) or TCI (Tokyo, Japan) and used as received without further purification. The NMR spectra were recorded on a Bruker (Billerica, MA, USA, 400 MHz) spectrometer by using tetramethylsilane (0 ppm for ^1^H and ^13^C NMR) as an internal standard. Absorption and fluorescence spectra were recorded on a Pekin Elmer Lambda 950 spectrophotometer (PerkinElmer, Waltham, MA, USA) and a Thermo Lumina spectrophotometer (Thermo Fisher Scientific Waltham, MA, USA), respectively, in a solution at room temperature (298 K) in a quartz cuvette of 1 cm path length. For the fluorescence lifetime measurements we used a FluoroCube spectrometer (HORIBA Jobin Yvon, Kyoto, Japan) equipped with a picosecond pulse laser and a picosecond photon detection module (TBX). The fluorescence quantum yield was recorded on a Hamamatsu absolute PL quantum yield spectrometer C11347-11. All calculations were performed with Spartan 2016 and the geometry of the molecule was optimized through density functional theory (DFT) calculations at the restricted B3LYP level with 6-31G*(d) basis set. X-ray diffraction data were collected at 298 K on a Bruker D8 Advance with Cu Kα radiation (Billerica, MA, USA). The elemental analyses were carried out on an Elementar vario MICRO cube. The thermal properties of samples were measured using TA DSC7020 (TA Instruments, New Castle, DE, USA) in differential scanning calorimetry (DSC) and TGA-Q50 in thermogravimetric analysis (TGA). Fourier transform infrared (FT-IR) spectra were recorded on a Nicolet 6700 spectrometer device (Thermo Fisher Scientific Waltham, MA, USA).

### 3.2. Materials and Synthesis 

1-bromo-4-hexylbenzene was purchased from Annaiji Chemical Company and used as received. 4,7-bis-(5-(trimethylstannyl) thiophene-2-yl) benzo[1,2,5]thiadiazole was purchased from Derthon Optoelectronic Materials Science Technology Co. ltd., (Shenzhen, China), and used as received. Other chemicals and solvents were commercially available and used as received unless otherwise claimed. The molecules were investigated by NMR, FTIR and elemental analyses (Figures S6–S11).

**4,7-bis(5-phenylthiophen-2-yl)benzo[c][1,2,5]thiadiazole (BTC0)**

^1^H NMR (CDCl_3_, 400 MHz, TMS, 298 K): *δ* 8.13 (d, *J* = 3.9 Hz, 2H), 7.91 (s, 2H), 7.72 (d, *J* = 7.7 Hz, 4H), 7.47–7.38 (m, 6H), 7.32 (t, *J* = 7.4 Hz, 2H).

^13^C NMR (CDCl_3_, 100 MHz, TMS, 298 K): *δ* 152.61, 145.62, 138.67, 134.13, 128.97, 128.63, 127.85, 125.85, 125.39, 124.11.

Analytically calculated for C_26_H_16_N_2_S_3_ (%): C, 68.99; H, 3.56; N, 6.19; S, 21.25. Found: C, 69.06; H, 3.37; N, 6.24; S, 21.48.

**4,7-bis(thiophene-2-yl)benzo[c][1,2,5]thiadiazole (BTC6)**

A 50 mL two-necked round bottom flask was charged with 4,7-Di(5-trimethyltin-thiophene)-2,1,3-benzothiadiazole (100 mg, 0.16 mmol), 1-Bromo-4-hexylbenzene (115 mg, 0.48 mmol), Pd_2_(dba)_3_ (14.65 mg, 0.016 mmol), P(o-toyl)_3_ (19.5 mg, 0.064 mmol), evacuated and back-filled with argon three times. Chlorobenzene (10 mL) was added via syringe. The reaction mixture was stirred at 110 °C for 24 h. Then, the solvent was evaporated under reduced pressure. Water was poured into the reaction mixture, extracted with DCM 3 times, the combined organic layer was washed with brine, and dried over MgSO_4_. The solvent was removed by evaporation under reduced pressure, and the resulting red solid was purified through column chromatography (silica gel, hexane: EtOAc = 5:1) to afford the target compound as red powder (95 mg, 96%).

^1^H NMR (CDCl_3_, 400 MHz, TMS, 298 K): *δ* 8.08 (s, 2H), 7.84 (s, 2H), 7.61 (d, *J* = 7.7 Hz, 4H), 7.36 (d, *J* = 3.8 Hz, 2H), 7.22 (d, *J* = 7.9 Hz, 4H), 2.64 (t, *J* = 7.5 Hz, 4H), 1.65 (q, *J* = 7.4 Hz, 4H), 1.42–1.23 (m, 12H), 0.94–0.86 (m, 6H).

^13^C NMR (CDCl_3_, 100 MHz, TMS, 298 K): *δ* 152.56, 138.16, 128.96, 128.63, 125.76, 125.24, 123.57, 35.71, 31.74, 31.38, 29.01, 22.63, 14.11.

Analytically calculated for C_38_H_40_N_2_S_3_ (%): C, 73.50; H, 6.49; N, 4.51; S, 15.49. Found: C, 73.46; H, 6.89; N, 4.58; S, 15.64.

## 4. Conclusions

In summary, we successfully designed and synthesized an intense fluorescence thermotropic liquid crystal material (BTC6). The hexyl chain attached to the terminus of the rigid chromophore was used to produce the liquid crystal feature. At the same time, the inclusion of alkyl chains was an effective strategy to induce the enhancement of the fluorescence quantum yield (*Φ*) in the solid state by weakening intermolecular π–π interactions. Moreover, the alkyl chain was able to promote the stability of electrochemical and thermal properties, which was beneficial to the device fabrication reproducibility and stability of the device performance.

## Figures and Tables

**Table 1:** The photophysical data of BTC0 and BTC6 in solution and powder at 298 K.

**Sample**	**Solution ^a^**							
	***λ*_abs_**	***λ*_em_**	**ε**	**Stokes Shift**	***Φ*_F_**	**<*τ*_f_> ^e,f^**	**k_r_^b^**	**k_nr_^c^**
**BTC0**	342 nm 489 nm	627 nm	5.0 × 10^4^ cm^−1^·M^−1^ 3.7 × 10^4^ cm^−1^·M^−1^	138 nm	0.81	7.83 ns	1.03 × 10^8^ s^−1^	2.43 × 10^7^ s^−1^
**BTC6**	348 nm 500 nm	637 nm	4.3 × 10^4^ cm^−1^·M^−1^ 3.2 × 10^4^ cm^−1^·M^−1^	137 nm	0.78	7.30 ns	1.07 × 10^8^ s^−1^	3.01 × 10^7^ s^−1^
**Sample**	**Powder**							
	***λ*_abs_**	***λ*_em_**	**Stokes Shift**	***Φ*_F_**	***τ*_f_ (f_i_) ^d^**	**<*τ*_f_> ^e,f^**	**k_r_^b^**	**k_nr_^c^**
**BTC0**	336 nm 473 nm	669 nm	196 nm	0.10	τ_1_ = 0.79 ns (15%) τ_2_ = 2.46 ns (77%) τ_3_ = 92.78 ns (8%)	1.97 ns	5.07 × 10^7^ s^−1^	4.57 × 10^8^ s^−1^
**BTC6**	346 nm 470 nm	678 nm	208 nm	0.26	τ_1_ = 6.33 ns (78%) τ_2_ = 13.36 ns (22%)	7.17 ns	3.63 × 10^7^ s^−1^	1.03 × 10^8^ s^−1^

^a^ In chloroform solution (1.0 × 10^−5^ M). ^b,c^ The radiative rate constant (k_r_) and the nonradiative rate constant (k_nr_) were calculated as follows: k_r_ = *Φ*_F_/<*τ*_f_> and k_nr_ = (1 − *Φ*_F_)/<*τ*_f_>. ^d^ The value in parentheses is the fractional contribution of component i to the total intensity, which was calculated by f_i_ = (A_i_τ_i_/∑A_i_τ_i_) × 100. ^e^ The averaged decay lifetime (<τ_f_>). ^f^ The averaged decay lifetime (<τ_f_>) was used to calculate the rate constants.

**Table 2:** The electrochemical HOMO-LUMO band gaps for BTC0 and BTC6.

Sample	*E*_Ox_^a^	*E*_Red_^b^	*E*_HOMO_^c^	*E*_LUMO_^d^	*E*_G_^e^
**BTC0**	1.08 V	−1.14 V	−5.40 eV	−3.18 eV	2.22 eV
**BTC6**	1.07 V	−1.16 V	−5.39 eV	−3.16 eV	2.23 eV

^a^ First oxidation potentials of the molecules by DPV. ^b^ First reduction potentials of the molecules by DPV. ^c^ The highest occupied molecular orbital energy calculated from E_HOMO_ = −(E_p_ + 4.8) eV, where E_p_ is the oxidation peak potential versus the Fc/Fc^+^ couple [34]. ^d^ The lowest unoccupied molecular orbital energy calculated from *E*_LUMO_ = −(E_n_ + 4.8) eV, where *E*_n_ is the reduction peak potential versus the Fc/Fc^+^ couple [34]. ^e^ Energy of the band gap calculated from the energy difference between the HOMO and LUMO.

## Supplementary Materials

The following are available online at https://www.mdpi.com/1996-1944/12/12/1919/s1, Figure S1: The absorption of solvatochromic effect for BTC6 (a) and BTC0 (b); The PL spectra of solvatochromic effect for BTC6 (c) and BTC0 (d). Figure S2: Transient PL decay curves of BTC0 and BTC6 in chloroform solutions (10^−6^ M) (a); Transient PL decay curves of BTC0 and BTC6 in solid state (b). Figure S3: Calculated electronic density contours and energy levels of HOMO and LUMO. Figure S4: Solution of BTC0 before electrochemical measurement (a) and after electrochemical measurement (b). Figure S5: TGA spectra of BTC0 and BTC6. Figure S6: ^1^H NMR of BTC0 in CDCl_3_. Figure S7: ^13^C NMR of BTC0 in CDCl_3_. Figure S8: ^1^H NMR of BTC6 in CDCl_3_. Figure S9: ^13^C NMR of BTC6 in CDCl_3_. Figure S10: FT-IR spectrum of BTC0. Figure S11: FT-IR spectrum of BTC6. Table S1: The absorption and PL maximum of solvatochromism.
