# Supplementary material for: Preparation of Luminescent Thermotropic Liquid Crystal from Benzodiathiazole Derivatives
Source: Materials (Basel). 2019 Jun 14;12(12):1919. doi: 10.3390/ma12121919 (PMC6630283; doi:10.3390/ma12121919)
Supplement: Supplementary file 1 [file materials-12-01919-s001.pdf]

materials

MDPI

Supplementary Materials

Preparation of Luminescent Thermotropic Liquid Crystal from Benzodiathiazole Derivatives

Yuchen Feng [1,3], Huijuan Yu [1,2], Dexun Xie [1,2,*], Yi Zhu [3], Xinhao Zhong [3], Chengjun Pan [3] and Guang Shao [1,2,*]

1 School of Chemistry, Sun Yat-sen University, Guangzhou 510275, China; Fyuchen96@163.com (Y.F.); yhjuan@mail.sysu.edu.cn (H.Y.)
2 Shenzhen Research Institute, Sun Yat-sen University, Shenzhen 518057, China
3 Shenzhen Key Laboratory of Polymer Science and Technology, College of Materials Science and Engineering, Shenzhen University, Shenzhen 518060, China; zhuyizhuyi913913@163.com (Y.Z.); s1920294@s.tsukuba.ac.jp (X.Z.); pancj@szu.edu.cn (C.P.)
* Correspondence: xiedx9@mail.sysu.edu.cn (D.X.); shaog@mail.sysu.edu.cn (G.S.)

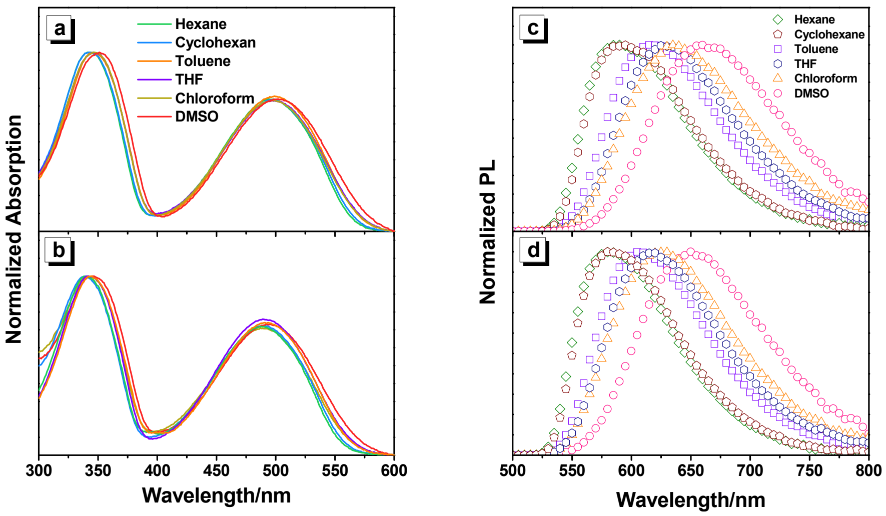

Figure S1. The absorption of solvatochromic effect for **BTC6** (a) and **BTC0** (b); The PL spectra of solvatochromic effect for **BTC6** (c) and **BTC0** (d).

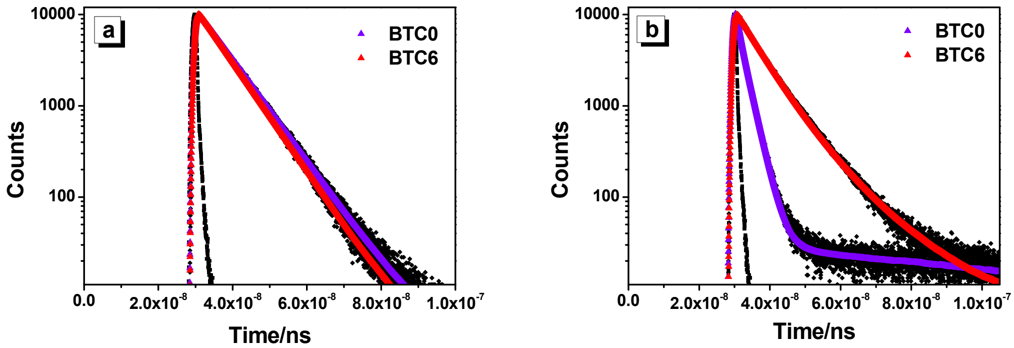

Figure S2. Transient PL decay curves of **BTC0** and **BTC6** in chloroform solutions (10^{-6} M) (a); Transient PL decay curves of **BTC0** and **BTC6** in solid state (b).

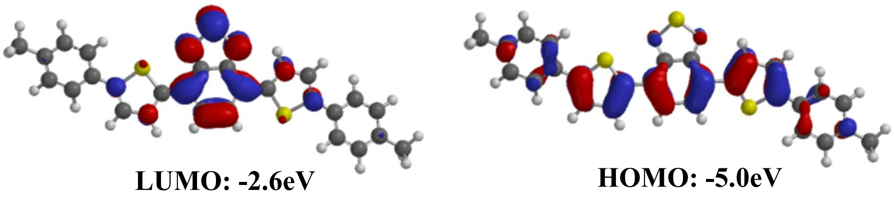

Figure S3. Calculated electronic density contours and energy levels of **HOMO** and **LUMO.**

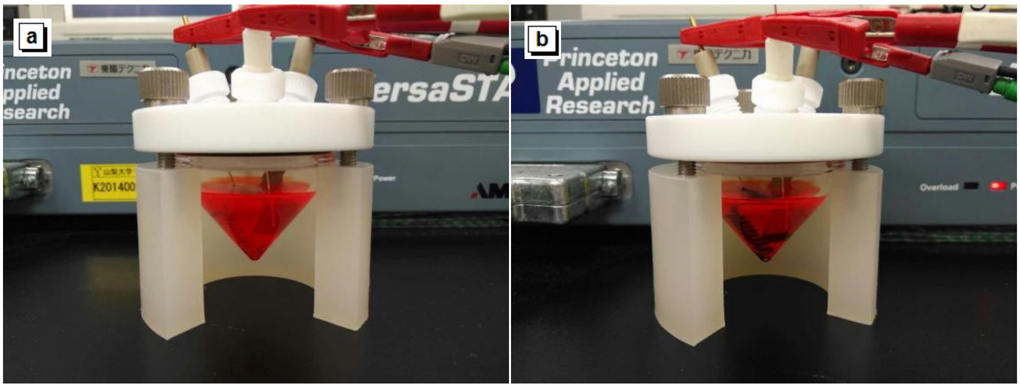

Figure S4. Solution of **BTC0** before electrochemical measurement (a) and after electrochemical measurement (b).

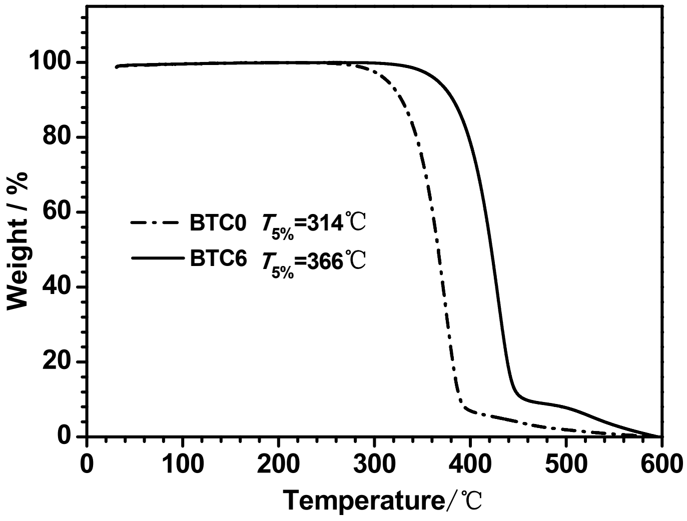

Figure S5. TGA spectra of BTC0 and BTC6.

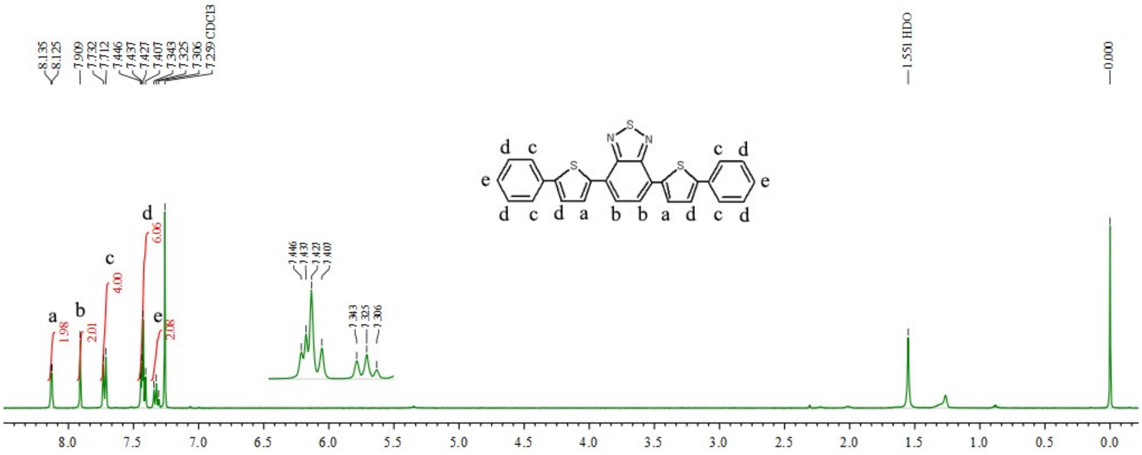

Figure S6. ^{1}H NMR of **BTC0** in $CDCl_3$.

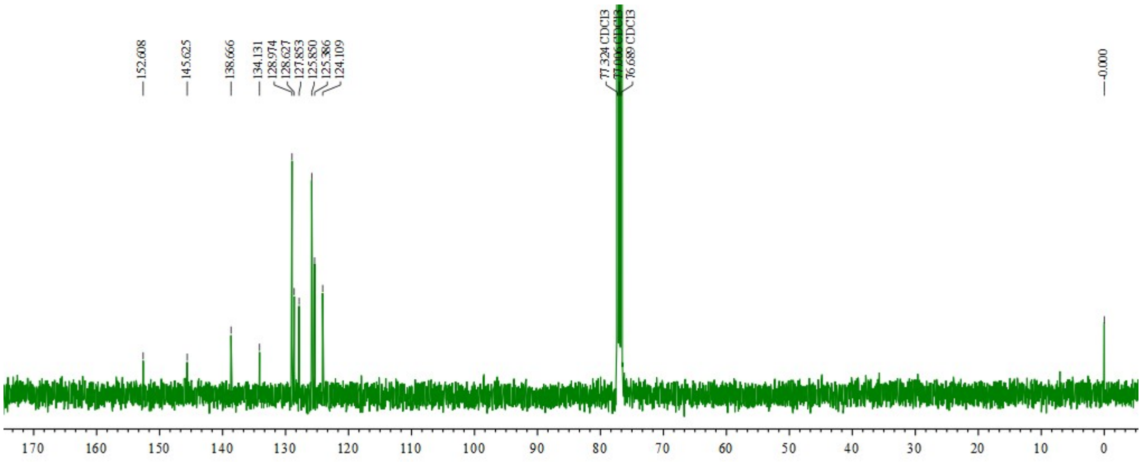

Figure S7. ^{13}C NMR of **BTC0** in $CDCl_3$.

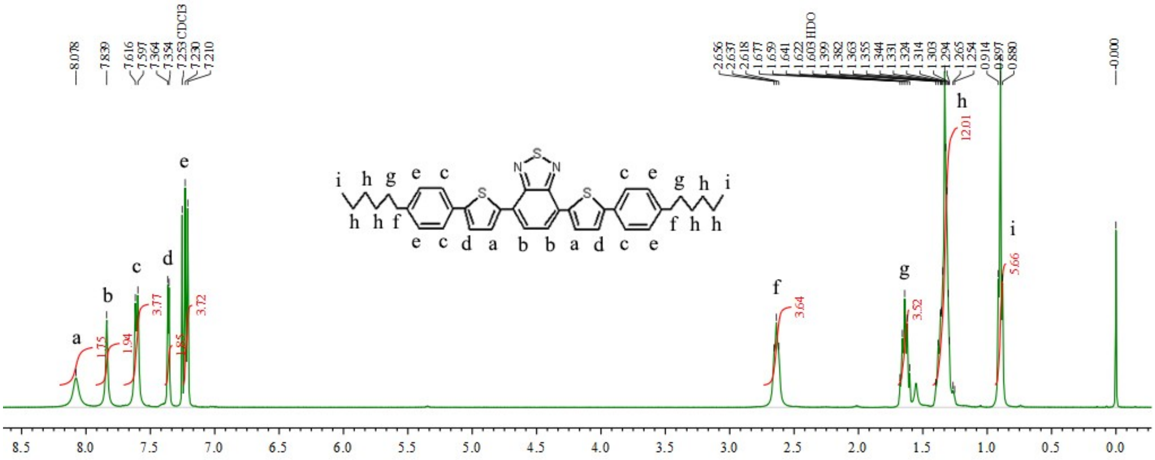

Figure S8. ^{1}H NMR of **BTC6** in $CDCl_3$.

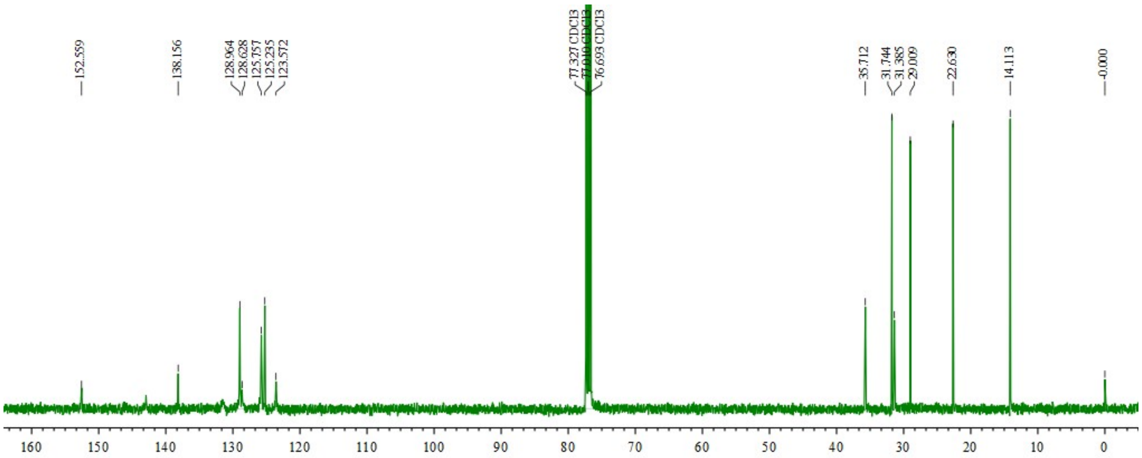

Figure S9. ^{13}C NMR of **BTC6** in $CDCl_3$.

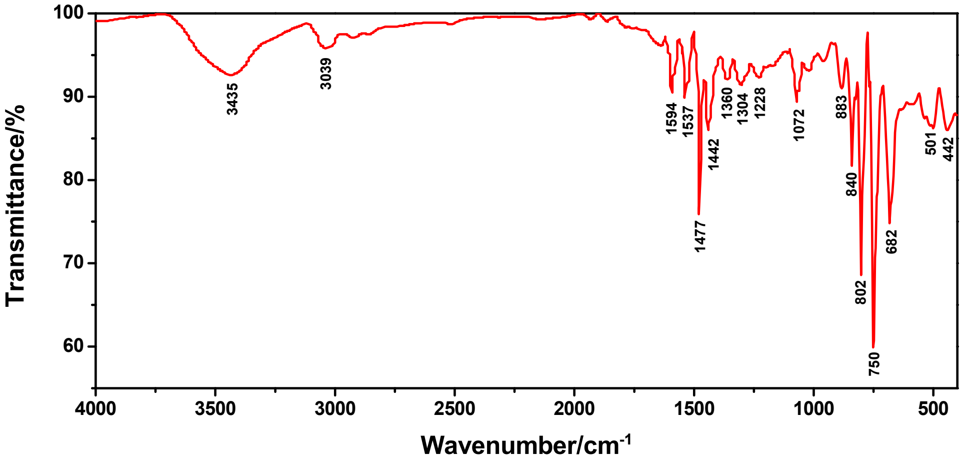

Figure S10. FT-IR spectrum of **BTC0.**

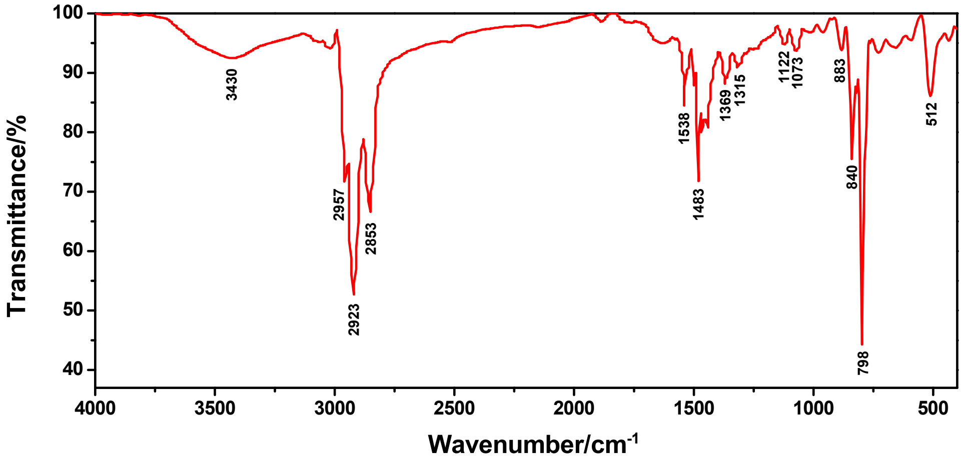

Figure S11. FT-IR spectrum of **BTC6.**

Table S1. The absorption and PL maximum of solvatochromism[a].

Solvent	ET(30)	BTC0		BTC6	
		λ_{abs}	λ_{em}	λ_{abs}	λ_{em}
Hexane	30.9	340 nm, 488 nm	582 nm	344 nm, 494 nm	587 nm
Cyclohexane	31.2	342 nm, 487 nm	579 nm	343 nm, 499 nm	594 nm
Toluene	33.9	341 nm, 492 nm	606 nm	346 nm, 499 nm	614 nm
THF	37.4	340 nm, 493 nm	618 nm	346 nm, 499 nm	626 nm
Chloroform	39.1	342 nm, 489 nm	627 nm	348 nm, 500 nm	637 nm
DMSO	45.0	345 nm. 492 nm	651 nm	351 nm, 502 nm	659 nm

[a] All the solutions are at concentration of 10^{-5} M.

© 2019 by the authors. Submitted for possible open access publication under the terms and conditions of the Creative Commons Attribution (CC BY) license (http://creativecommons.org/licenses/by/4.0/).
